# Mutation in the Gene Encoding Ubiquitin Ligase LRSAM1 in Patients with Charcot-Marie-Tooth Disease

**DOI:** 10.1371/journal.pgen.1001081

**Published:** 2010-08-26

**Authors:** Duane L. Guernsey, Haiyan Jiang, Karen Bedard, Susan C. Evans, Meghan Ferguson, Makoto Matsuoka, Christine Macgillivray, Mathew Nightingale, Scott Perry, Andrea L. Rideout, Andrew Orr, Mark Ludman, David L. Skidmore, Timothy Benstead, Mark E. Samuels

**Affiliations:** 1 Department of Pathology, Dalhousie University, Halifax, Nova Scotia, Canada; 2 Maritime Medical Genetics Service, Izaak Walton Killam Health Centre, Halifax, Nova Scotia, Canada; 3 Department of Ophthalmology and Visual Sciences, Dalhousie University, Halifax, Nova Scotia, Canada; 4 Department of Pediatrics, Division of Medical Genetics, Izaak Walton Killam Health Centre and Dalhousie University, Halifax, Nova Scotia, Canada; 5 Department of Medicine, Division of Neurology, Dalhousie University, Halifax, Nova Scotia, Canada; 6 Centre de Recherche de l'Hôpital Ste-Justine, Université de Montréal, Montréal, Quebec, Canada; University of Minnesota, United States of America

## Abstract

Charcot-Marie-Tooth disease (CMT) represents a family of related sensorimotor neuropathies. We studied a large family from a rural eastern Canadian community, with multiple individuals suffering from a condition clinically most similar to autosomal recessive axonal CMT, or AR-CMT2. Homozygosity mapping with high-density SNP genotyping of six affected individuals from the family excluded 23 known genes for various subtypes of CMT and instead identified a single homozygous region on chromosome 9, at 122,423,730–129,841,977 Mbp, shared identical by state in all six affected individuals. A homozygous pathogenic variant was identified in the gene encoding leucine rich repeat and sterile alpha motif 1 (LRSAM1) by direct DNA sequencing of genes within the region in affected DNA samples. The single nucleotide change mutates an intronic consensus acceptor splicing site from AG to AA. Direct analysis of RNA from patient blood demonstrated aberrant splicing of the affected exon, causing an obligatory frameshift and premature truncation of the protein. Western blotting of immortalized cells from a homozygous patient showed complete absence of detectable protein, consistent with the splice site defect. LRSAM1 plays a role in membrane vesicle fusion during viral maturation and for proper adhesion of neuronal cells in culture. Other ubiquitin ligases play documented roles in neurodegenerative diseases. LRSAM1 is a strong candidate for the causal gene for the genetic disorder in our kindred.

## Author Summary

Sensory motor neuropathies are diseases of the peripheral nervous system, involving primarily the nerves which control our muscles. These can result from either genetic or non-genetic causes, with genetic causes usually referred to as Charcot-Marie-Tooth (CMT) disease after the three clinicians who first described the key diagnostic markers. CMT patients lose muscle function, mainly in their arms and legs, with increasing severity during their lives. There are almost two dozen known genes that can mutate to cause CMT, and these fall into a wide variety of biochemical cellular pathways. We identified a group of patients with CMT from a small rural community, with good reason to suspect a genetic basis for their disease. Using high-throughput mapping and DNA sequencing technologies developed as part of the Human Genome Project, we were able to find the likely mutated gene, which was not any of the previously known CMT genes. Based on its sequence, the gene, called LRSAM1, probably plays a role in the correct metabolism of other proteins in the cell. Among the known CMT genes, some are also involved in protein metabolism, suggesting that this is a generally important pathway in the neurons that control muscle activity.

## Introduction

Charcot-Marie-Tooth disease (CMT) comprises a set of genetically heterogeneous disorders of the peripheral nervous system, affecting motor and sensory function. CMT is the most common inherited neuromuscular disorder, with a wide range of clinical presentations, but as described by OMIM (118200), the salient features of CMT include a slowly progressive weakness and atrophy of the musculature, predominantly of the distal lower limb. This weakness often affects the patients ability to walk or run, and eventually can progress to reach the upper extremity. Within the broad group of patients defined clinically, there are various categories of CMT defined by neurophysiological subphenotypes, pathological findings on biopsy, modes of familial transmission, and specific mutated genes identified in individual patients. These criteria have been extensively reviewed in recent literature [1]–[17]. A query of OMIM for genes causing Charcot-Marie-Tooth yields 26 separate entries with allelic variants; the database of inherited peripheral neuropathies notes 31 gene entries for CMT plus an additional 7 described as causing distal hereditary motor neuropathy. Nonetheless, mutations in new genes associated with CMT continue to be reported[18].

The functions of genes whose mutation yields a CMT or closely related motor neuropathy phenotype span a wide range of disparate biochemical activities including structural components of myelin (PMP22, P0), a mitochondrial transport and fusion protein (MFN2), transcription factors (SOX, EGR2), components of protein degradation pathways (DNM2, RAB7, LITAF), tRNA synthetases (GARS, YARS), a nuclear structural component (LMNA) and others [19]. Thus, novel CMT genes are difficult to predict through selection of biological candidates for sequencing in unexplained patients. The best approach for identifying the genetic cause of unexplained CMT remains linkage mapping in multiplex families, with adequate statistical power dependent on the mode of transmission, the specifics of pedigree and local population structure.

We report the mapping of a novel form of autosomal recessive axonal CMT through homozygosity mapping in an extended consanguineous pedigree of a local founder population. The identified gene appears to play a role in vesicle metabolism, consistent with some other CMT genes.

## Results/Discussion

In the course of clinical work, we ascertained a patient with Charcot-Marie-Tooth disease, most closely similar to subtype AR-CMT2 (recessive, axonal), although this clinical presentation has sometimes been included as a type of CMT4[16]. The index patient noted the gradual onset of weakness around age 20, particularly affecting his distal lower extremities, but also present in the hands. He noted episodic muscle cramping of extremity and trunk muscles. He lost the ability to run in his early 20s. He denied sensory symptoms. He had erectile dysfunction and urgency of urination, but no other autonomic symptoms or evidence of spasticity. At the time of examination he demonstrated bilateral *pes cavus*, with marked wasting of distal lower extremity muscles and mild wasting of hand intrinsic muscles. Fasciculations were present in upper and lower extremity muscles. In the lower extremities he had grade 4 out of 5 ankle dorsiflexion strength (MRC scale), grade 4 hand intrinsic muscle strength and other muscles were grade 5. He could not walk on either the toes or heels. There was no gait ataxia. Upper and lower extremity tendon reflexes were absent. He had mild loss of sensation on the fingertips and severe loss of sensation in the feet and legs, most markedly to vibration, but also involving proprioception and pain perception. Laboratory investigation demonstrated an elevated serum creatine kinase (CK) from 1082 to 1921 U/L (18-199 U/L). Nerve conduction studies and needle electromyography demonstrated a diffuse sensorimotor peripheral neuropathy. There was no evidence of a primary muscle disorder. The predominant electrophysiological pattern was consistent with axonal degeneration (see Table S1). Sensory nerve action potentials were small or absent. All of the upper extremity motor nerve conduction velocities were faster than 38 m/s. The ulnar compound muscle action potential amplitude was small and a repeat study 2 years later demonstrated both median and ulnar compound muscle action potential amplitudes were small with normal motor conduction velocities. These are accepted criteria for an axonal CMT [1]. Upper and lower extremity muscles demonstrated ample denervation and partial reinnervation, with fibrillation and reduced recruitment of large motor unit potentials. Denervation of paraspinal muscles indicated axonal degeneration was present at very proximal nerve levels. Temporal dispersion was seen in some motor nerve conductions, but no conduction block, which may be an indication of an element of secondary demyelination, but the predominant electrophysiologic pattern was axonal.

**Figure 1:**
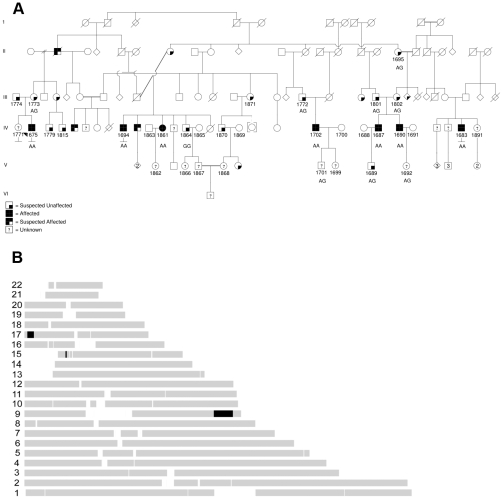
Maritime family with Charcot-Marie-Tooth and genetic mapping. (A) Pedigree, with affected patients shaded in black, sampled individuals have four digit id numbers directly below symbols, proband 1675 is indicated with black arrowhead. Sequenced individuals have mutation status indicated (wild type is GG, homozygous mutant is AA, heterozygous mutant is AG). (B) Homozygous haplotype (HH) analysis. Map of the RCHH intervals shared by 5 patients identified by Homozygosity Haplotype algorithm with a cutoff of 3.0 cM.

The proband is a member of an extended multiply consanguineous family derived from a rural eastern Canadian population isolate; the extended pedigree includes five additional affected individuals with similar suites of symptoms (Figure 1A). Other affected family members exhibited sensory and motor dysfunction with pes cavus. Autonomic symptoms have not been consistently reported. Weakness and wasting has usually been moderate and predominantly in distal lower extremity muscles. The onset of symptoms has usually been in early adult years. One patient was not aware of any difficulties, but had examination abnormalities in his 40's. Some of the affected individuals are able to ambulate into later years, though others have become wheelchair dependent. Sensory symptoms are sometimes not reported, but sensory examination is consistently markedly abnormal, with loss of vibration sense often up to proximal legs and hips. Proprioception loss has been severe in some affecteds with accompanying sensory ataxia. Laboratory abnormalities that are available in only a few patients include mild increased CSF protein and increased serum CK. One patient had significant essential tremor, but that has not usually been reported. When EMG data is available, the pattern is typically predominantly axonal degeneration with only mildly slowed or normal motor nerve conduction velocities and no upper extremity motor nerve conduction velocities slower than 38 m/s. One other patient had evidence of paraspinal muscle denervation, with a normal MRI of the spine, suggesting axonal degeneration at very proximal nerve levels from the neuropathy.

Based on transmission of the trait in the pedigree, the genetics are consistent with an autosomal recessive disorder. Given the isolation of the regional population, it seemed likely that all affected individuals in our cohort might be homozygous for the same causal mutation, sharing a chromosomal haplotype around the causal gene. We sampled DNA from six affected patients and related family members. We performed high density genome-wide SNP genotyping of five affected individuals. Formal linkage analysis using a recessive model was not deemed useful, given the highly consanguineous pedigree structure and also the impossibility of obtaining reliable marker allele frequencies for this small subpopulation. Instead, we used the homozygous haplotype (HH) method to test for linkage to any of 23 known relevant CMT loci. The HH method is a rapid non-parametric algorithm that utilizes the subset of completely homozygous markers in samples from affected individuals, and looks for consistent loci by excluding regions where affected individuals are homozygous for different alleles of a given SNP [20], [21]. The method is robust due to the high density of commercial genotyping panels. In this case, HH confidently excluded all of the known relevant CMT loci, under the assumption that all five affected individuals in our pedigree are homozygous for the same causal allele. HH flagged three chromosomal regions as potentially linked, on chromosomes 9, 15 and 17 (Figure 1B).

Subsequently we genotyped additional pedigree members including one more affected, and looked for regions of extended homozygosity shared identical-by-state (IBS) in the six affected individuals but not in unaffecteds. As shown in Table 1, among the longest series of consecutive homozygous SNPs, a region on chromosome 9 appeared as a clear outlier. This region corresponds to that predicted from HH analysis, and extends from rs1324475 at 122,423,730 Mbp to rs10987845 at 129,841,977 Mbp. It is interrupted by several single heterozygous SNPs, mostly in one particular sample; these presumably represent false heterozygote genotype calls. In contrast, the potential regions found by HH on chromosomes 15 and 17 were not homozygous in all six affected individuals when all marker data was considered. The likely linked interval is 7.42 Mbp in size on chromosome 9, and includes 84 RefSeq annotated genes, including a cluster of 14 olfactory receptor genes which were not considered likely candidates.

**Table 1:** All intervals of 35 or more consecutive SNPs homozygous and identical by state among the six affected CMT samples.

SNPs	Chr	StartSNP	EndSNP	Start(bp)	End(bp)	Size(bp)
***378***	***9***	***rs2479106***	***rs10123453***	***125 565 033***	***127 899 007***	***2 333 975***
***337***	***9***	***rs9409287***	***rs7039798***	***128 044 215***	***129 597 047***	***1 552 833***
***219***	***9***	***rs4837971***	***rs10986087***	***124 289 305***	***125 482 561***	***1 193 257***
***177***	***9***	***rs1324475***	***rs10760198***	***122 423 730***	***123 650 357***	***1 226 628***
***146***	***9***	***rs10760198***	***rs4837971***	***123 650 357***	***124 289 305***	***638 949***
***67***	***9***	***rs10123453***	***rs9409287***	***127 899 007***	***128 044 215***	***145 209***
57	18	rs17240415	rs3891810	64 801 868	64 929 306	127 439
49	21	rs8132309	rs363568	29 767 744	29 954 834	187 091
46	4	rs17353301	rs10517306	33 489 284	34 049 422	560 139
41	7	rs4646450	rs2246709	99 104 254	99 203 655	99 402
***40***	***9***	***rs7039798***	***rs10987845***	***129 597 047***	***129 841 977***	***244 931***
37	1	rs6660164	rs4310401	80 222 072	80 428 702	206 631
36	9	rs11787664	rs10118040	116 821 477	116 919 235	97 759
35	4	rs4696998	rs7655220	21 588 415	21 746 131	157 717

Intervals are in descending order of number of consecutive SNPs, although the chromosome 9 italicized regions are contiguous.

**Figure 2:**
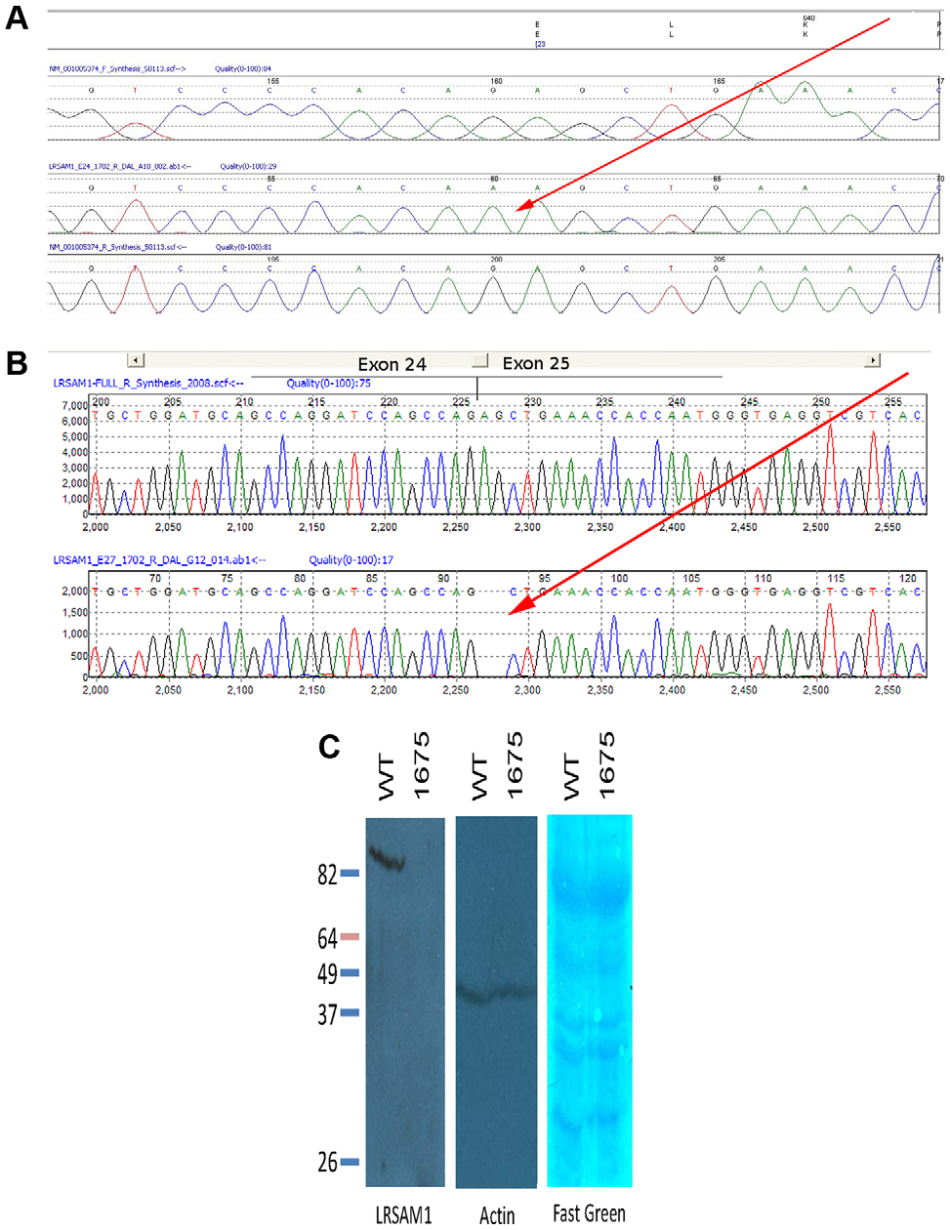
Sequence showing mutation in genomic and cDNA of affected patient. (A) Mutation of splice acceptor site AG of LRSAM1 exon 24 (25 in alternative isoform 3) to dinucleotide AA in genomic DNA of patient 1702. Upper to lower panels: translation of coding exon; virtual chromatogram of consensus genomic sequence forward direction; sequence chromatogram of affected patient reverse direction; virtual chromatogram of consensus genomic sequence reverse direction. Red arrow points to homozygous mutation. (B) Sequence of cDNA from RNA of patient 1702 showing aberrant splice site utilization and frameshift of encoded protein. Upper panel, sequence chromatogram of correctly spliced cDNA from exon 24 to 25 (per isoform 3); lower panel, sequence chromatogram of incorrectly spliced cDNA from affected patient. Two base deletion caused by splicing interior to exon 25 (red arrow). (C) Western blot of LRSAM1 protein in cells cultured from patient homozygous for LRSAM1 mutation. EBV-transformed B- lymphocytes from control or patient 1675 were extracted and Western blotted with anti-LRSAM1 antibody. Left, anti-LRSAM1; center, anti-actin; right, Fast green total protein stain.

We prioritized genes likely to have neuronal or neuromuscular function based on manual review. In all we sequenced 314 coding exons of 18 genes (HSPA5, DENND1A, RABGAP1, RAB14, STXBP1, DNM1, SPTAN1, DAB2IP, LHX2, TOR1A, GSN, LHX6, LMX1B, CDK9, CDK5RAP2, FPGS, SH2D3C, LRSAM1), until we observed a particular homozygous variant in the gene LRSAM1 (Figure 2A). This variant changes a coding exon consensus splice acceptor AG dinucleotide to an AA. There are three RefSeq annotated isoforms of LRSAM1, differing in the 5′ noncoding region, generating transcripts of either 25 or 26 exons. All three splice forms predict the same open reading frame; the variant identified in our patients lies in the penultimate coding exon, either 24 (isoforms 1, 2) or 25 (isoform 3). The variant was found homozygous in all six affected individuals, and either wild type or heterozygous as expected among sequenced parents and siblings (Figure 1A). This variant is not present in dbSNP build 130 which includes 2 million novel SNPS recently submitted by the 1000 Genomes project, nor was it detected in any of 150 local control (a mix of anglo- and franco-phonic individuals) or 96 CEPH Caucasian control samples, totalling almost 500 control chromosomes. No other homozygous coding variants were detected by sequencing this set of candidate genes.

The variant in question changes the consensus splice acceptor site. We tested three splice site prediction programs (Berkeley Drosophila Project, NetGene2 and SplicePort) to see whether they were sensitive to the alternative site used in the homozygous patients. All three programs correctly predicted the *bona fide* splice acceptor site in the wild type sequence. The Berkeley tool failed to predict the alternative AG two nucleotides internally in the mutant sequence, while NetGene2 and SplicePort predicted this acceptor site though with low confidence. We were able to test directly whether splicing of the exon was altered, using total RNA extracted from a fresh blood sample from one affected patient (1702). By qualitative RT-PCR, we saw a product of the appropriate size in both a control sample and the affected patient sample, at roughly equivalent intensities (*d.n.s.*) Although the resolution of the electrophoresis was much less than single nucleotide, sequencing of the sample product from the affected patient showed that splicing was to the next AG directly following the true acceptor site, two bases into penultimate exon 24 (or 25 as per isoform 3) (Figure 2B). This causes an obligatory frameshift, leading to an altered open reading frame and premature truncation of the protein after 643 (out of 723) residues in all three spliced isoforms. The effect of this change on protein expression was tested directly by Western blot using EBV-transformed B-lymphocyte cell lines (B-LCL) derived from a healthy control and from one of the affected CMT patients. While a single strong band was detected by the anti-LRSAM1 antibody in the control B-LCL (molecular weight approximately 78 kDa), no protein was detected in the B-LCL derived from the CMT patient (Figure 2C). Either the truncated protein is rapidly degraded, or else is rendered non-reactive with our antibody. In either case, the result is most likely to be a significant loss-of-function of the gene product, although unusual gain-of-function effects of a truncated protein can be imagined (though these might be expected to behave in a dominant not recessive fashion).

LRSAM1, leucine rich repeat and sterile alpha motif containing 1, is predicted to be an E3 type ubiquitin ligase [22]. It is also known as TAL (TSG101-associated ligase) and RIFLE. TSG101 itself is a tumor suppressor gene, with a reported role in maturation of human immunodeficiency virus, and LRSAM1 is implicated in its metabolism directly by polyubiquitination. TSG101 is involved in retroviral vacuolar budding. Interestingly, another TSG101-ubiquitinating ligase is known, (Mahogunin, or MGRN1), for which knockout mice exist and exhibit a neurodegenerative phenotype. Moreover, the known CMT gene LITAF, also called SIMPLE, interacts with mouse ubiquitin ligase gene product NEDD4 [23], also potentially with TSG101 [24], and may itself be an E3 ubiquitin ligase [25], These related findings support the interpretation that mutation of LRSAM1 is probably causal in our patients. It remains to be determined whether the pathogenic effects of mutations in these protein degradation pathway genes act directly via specific neuron-specific proteins (such as PMP22) or more generally through decreasing cell viability.

The currently recommended diagnostic paradigm for Charcot-Marie-Tooth entails a complex flow chart combining clinical, familial and molecular genetic analyses [3]. While this approach makes sense when DNA sequencing technologies are cost-limiting, this mixed paradigm could soon be replaced by a more comprehensive and pre-emptive molecular analysis. With the advent of whole genome reagents such as all-exon hybridization capture oligonucleotide libraries, together with the tremendous cost-reductions in DNA sequencing using next-generation nanotechnologies, it should soon be feasible to sequence either entire patient genomes, or entire exomes, for less than the cost of traditional Sanger-based fluorescent capillary sequencing of sets of candidate genes [26]–[28]. We envisage an analysis paradigm whereby all patients with a potential genetic diagnosis, across any medical subdiscipline, may first be sequenced to identify likely pathogenic variants, which can then be cross-indexed with clinical parameters to flag likely causal genes. This approach has recently been shown to be feasible in a research context, including detection of a pathogenic variant in a family segregating a known form of CMT [29]–[32].

## Materials and Methods

### Clinical ascertainment and consent

Approval for the research study was obtained from the Capital Health research ethics board. Patients were identified in the course of routine clinical ascertainment and treatment of movement disorders in the neurology clinic at the Halifax Infirmary. All sampled family members provided informed consent to participate in the study. DNA was obtained from blood samples using routine extraction methods.

### Genotyping and analysis

Whole-genome SNP scanning was performed at the McGill University and Genome Quebec Centre for Innovation, using the Illumina Human610-Quadv1_B panel. Data were scanned using the Bead Array Reader, plate Crane Ex, and Illumina BeadLab software, on Infinium II fast scan setting. Allele calls were generated using Beadstudio version 3.1 with genotyping module. Data are generated in three different output formats, AB, Forward strand, and Top strand (as defined by Illumina). We used AB format for all linkage analyses.

Homozygosity haplotype (HH) analysis was performed according to the method of Miyazawa [21]. The source code of HH program was modified to customize the format of output. The parameter LARGEGAP defined in the header file, which is used to define large gap of two consecutive SNPs like centromere, was changed from the default value 300,000 bp to 400,000 bp to accommodate some non-centromere spaces for HumanHap610 genotypes. The revised C source code of HH program was compiled with GNU compiler on a Linux-based operating system Fedora. HH analysis requires a SNP annotation file, which includes SNP name, physical coordinates, genetic distances, and minor allele frequencies. The SNP annotation file provided by HH software is for the Affymetrix 500K GeneChips Human Mapping Array Set. The HH format annotation of Illumina HumanHap610 for CEPH population was created from the SNP annotations downloaded from Illumina website. The genetic distances of SNPs with empty value, inconsistent value, or zero were interpolated according to the physical coordinates of their flanking SNPs. HH analysis was performed with a cutoff value 3.0 cM. Homozygosity analysis was performed using customized scripts and manual inspection comparing samples from affected and unaffected pedigree members.

### Mutation detection and analysis

Annotated coding exons were amplified by PCR using standard methods, and sequenced at Dalhousie University, using Sanger fluorescent sequencing and capillary electrophoresis. Sequence traces were analyzed using MutationSurveyor (Soft Genetics, Inc.) Specific primers for amplification of LRSAM1 exons and PCR conditions are provided in Table S2.

### Western blot

EBV-transformed B-LCL cells derived from a healthy subject or CMT patient 1675 were cultured in RPMI with 10% FBS and 1% pen/strep in 5% CO2. Cells were pelleted and lysed in lysis buffer (50 mM Tris-HCL, pH 7.4, 150 mM NaCl, 2 mM EDTA, 0.2% Triton X-100 with 1 mM PMSF and protease inhibitor tablet (Sigma) added to ice cold buffer immediately prior to use). Cells were broken by vortexing for 1 minute. Cell debris was removed by centrifugation at 16000×g for 10 minutes. Protein concentration was determined by the Bradford method (Sigma). Samples were diluted to 6 microg/microL in lysis buffer, then to 2 microg/microL in sample dye (125 mM Tris-HCL ph 6.8 with 20% glycerol, 4% SDS, 0.04% bromophenol blue, 10% 2-mercaptoethanol). Samples were heated to 95°C for 5 minutes prior to separation of 50 ug sample on a 7.5% SDS-PAGE gel. Benchmark pre-stained protein ladder (Invitrogen) was included on the gel. Protein was transferred by wet transfer to methanol-wetted PVDF membrane in transfer buffer (25 mM Tris-base, 192 mM glycine). Membranes were blocked overnight in blocking buffer (5% skim milk powder, 0.05% Tween 20, in PBS pH 7.4). Anti-LRSAM1 antibody (abcam) diluted 1∶500 in blocking buffer was incubated overnight at 4 degrees. Blots were washed in PBS- (0.05% Tween 20 in PBS pH 7.4) 15 minutes plus 3×5 minutes. HRP labelled secondary anti-mouse antibody, diluted 1∶2500 in blocking buffer, was incubated for 1 hour at room temperature. Blots were washed as above. HRP was visualized using SuperSignal West Pico Substrate (Fisher Scientific) and exposing to X-ray film for 3-5 minutes. Protein transfer to the gel was confirmed by staining the PVDF membrane with Fast Green.

The URLs for the data and analytic approaches presented herein are as follows:

Online Mendelian Inheritance in Man (OMIM), http://www.ncbi.nlm.nih.gov/Omim/

UCSC Genome Browser, http://genome.ucsc.edu/

NCBI, http://www.ncbi.nlm.nih.gov/

Database of inherited peripheral neuropathies, http://www.molgen.ua.ac.be/CMTMutations/Home/Default.cfm

## Supporting Information

Table S1 Nerve conduction study of proband. Normal values in brackets. Abbreviations: NR (not recordable), EDB (extensor digitorum brevis), AH (abductor hallucis), APB (abductor pollicus brevis), ADM (abductor digiti minimi).
(0.02 MB DOC)

Table S2 LRSAM1 PCR primers and conditions. 95C for 2 min. Followed by 25 cycles of 95C for 30 sec, the appropriate annealing temperature (listed in table above) for 30 sec, and 72C for 1 min. Finish with 72C for 5 min.
(0.06 MB DOC)
