# Supplementary material for: Mutation in the Gene Encoding Ubiquitin Ligase LRSAM1 in Patients with Charcot-Marie-Tooth Disease
Source: PLoS Genet. 2010 Aug 26;6(8):e1001081. doi: 10.1371/journal.pgen.1001081 (PMC2928813; doi:10.1371/journal.pgen.1001081)
Supplement: Table S1 — Nerve conduction study of proband. Normal values in brackets. Abbreviations: NR (not recordable), EDB (extensor digitorum brevis), AH (abductor hallucis), APB (abductor pollicus brevis), ADM (abductor digiti minimi). (0.02 MB DOC) [file pgen.1001081.s001.doc]

**Table S1 Nerve Conduction Study of proband**

Nerve Recording site Amplitude Distal latency (ms) Conduction velocity (m/s)

Ulnar motor ADM 4.1 mV (>6) 2.7 (<3.6) 56 (>51)

Median motor APB 4.9 mV (>4) 4.4 (<4.5) 53 (>48)

Ulnar sensory Digit V 5.2 μV (>9) 3.1 (<3.1) 61 (>55)

Median sensory Digit II 4.9 μV (>15) 3.3 (<3.6) 63 (>55)

Peroneal motor EDB 3.2 mV (>2) 6.0 (<6.6) 36 (>40)

Tibial motor AH 1.1 mV (>4) 4.5 (<5.9) 45 (>40)

Sural sensory Lateral malleolus NR
