# Supplementary material for: Mutation in the Gene Encoding Ubiquitin Ligase LRSAM1 in Patients with Charcot-Marie-Tooth Disease
Source: PLoS Genet. 2010 Aug 26;6(8):e1001081. doi: 10.1371/journal.pgen.1001081 (PMC2928813; doi:10.1371/journal.pgen.1001081)
Supplement: Table S2 — LRSAM1 PCR primers and conditions. 95C for 2 min. Followed by 25 cycles of 95C for 30 sec, the appropriate annealing temperature (listed in table above) for 30 sec, and 72C for 1 min. Finish with 72C for 5 min. (0.06 MB DOC) [file pgen.1001081.s002.doc]

Table S2 LRSAM1 PCR primers and conditions

| CCCTAACTTCTCTCCTGTGCC | LRSAM1_E02_F (First coding exon) |
| --- | --- |
| CTACTCCATGGGGACCGAG | LRSAM1_E02_R 60C |
| tatgttctttgCCTGGCTTG | LRSAM1_E03_F |
| GATCCAAGAAAACGGGACAG | LRSAM1_E03_R 55C |
| CCTGCCTGTCTCTTCCTCAC | LRSAM1_E04_F |
| TCAAAGTCCCTTGTGGGTTC | LRSAM1_E04_R 60C |
| CTCCCACGCCCTTAGCC | LRSAM1_E05_F |
| CTCTGACATGCAAGAGGCAG | LRSAM1_E05_R 60C |
| CCGGAGTCTGAGGGACTTTC | LRSAM1_E06_F |
| AGCCTGCTTCCACTGGC | LRSAM1_E06_R 60C |
| ttgaccttgtgatccacctg | LRSAM1_E07_F |
| CTGCCCTCCTGCTCAATG | LRSAM1_E07_R 60C |
| CAGGAAGCTGGTGATGGG | LRSAM1_E08_F |
| TAATGTGGGAGAGAGGGGAG | LRSAM1_E08_R 60C |
| aaggaaatcgtgtggtctcc | LRSAM1_E09_F |
| tgtggccatttctgtctctg | LRSAM1_E09_R 60C |
| TGGGTACCTCAGCTGTCTCC | LRSAM1_E10_F |
| atacatgcacacgcacacac | LRSAM1_E10_R 60C |
| ggagaaccactgCTGCC | LRSAM1_E11_F |
| GACCTCTAATTCTGAGCTGGG | LRSAM1_E11_R 60C |
| TTCTCCCGACTTCTGTGTCC | LRSAM1_E12_F |
| CAGGAGGCCACTGGCTG | LRSAM1_E12_R 60C |
| agtcagtacccCTCACGGC | LRSAM1_E13_F |
| ACACTCCTCCCGGCTCC | LRSAM1_E13_R 55C |
| gaacccagagcttcaaggtg | LRSAM1_E14_F |
| GTCCTGGCATAATCCTCTGG | LRSAM1_E14_R 55C |
| gggattccctgttggaaatg | LRSAM1_E15_F |
| GGCCAACAGAGGGAGGG | LRSAM1_E15_R 60C |
| TCAGTTCCTGTGGAAATCCC | LRSAM1_E16_F |
| GGAGGTTTAGTGCCCAGCTC | LRSAM1_E16_R 60C |
| tagtaggtgctcgggaaacg | LRSAM1_E17_F |
| GTGTGGAGCTCTGCACTCTG | LRSAM1_E17_R 60C |
| TTGCTTTTCACAGGGATGG | LRSAM1_E18_F |
| CTGGAGGGCCACTCTGC | LRSAM1_E18_R 60C |
| GCAGCACAAAACTGAACTGTG | LRSAM1_E19_F |
| CTGGGAGGGAGCACCAG | LRSAM1_E19_R 60C |
| AGGGCCTCTTAGCTTTGTCC | LRSAM1_E20_F |
| GGCAGGAGATAACGCTTGG | LRSAM1_E20_R 55C |
| CAAACCCTTCATTTCCTGTTG | LRSAM1_E22_F |
| CTGGTCAGGACAGCGGC | LRSAM1_E22_R 55C |
| ACGTGGCTCACACCATTTAG | LRSAM1_E23_F |
| CCCACCAGAGCCCACAC | LRSAM1_E23_R 60C |
| CCAGGGGTTAGGGTCAGC | LRSAM1_E24_F |
| CAGAGACAACCCTGTACCTGC | LRSAM1_E24_R 60C |
| CCTGGGACTCCTGGAACC | LRSAM1_E25_F |
| GCTGGCACAAGGCTGAG | LRSAM1_E25_R 60C |
| attgggcagagaaccgagt | LRSAM1_E21_F |
| caggtgtttctaaaaatggcaag | LRSAM1_E21_R 55C |
